# Migratory birds modulate niche tradeoffs in rhythm with seasons and life history

**DOI:** 10.1073/pnas.2316827121

**Published:** 2024-09-23

**Authors:** Scott W. Yanco, Ruth Y. Oliver, Fabiola Iannarilli, Ben S. Carlson, Georg Heine, Uschi Mueller, Nina Richter, Bernd Vorneweg, Yuriy Andryushchenko, Nyambayar Batbayar, Mindaugas Dagys, Mark Desholm, Batbayar Galtbalt, Andrey E. Gavrilov, Oleg A. Goroshko, Elena I. Ilyashenko, Valentin Yu Ilyashenko, Johan Månsson, Elena A. Mudrik, Tseveenmyadag Natsagdorj, Lovisa Nilsson, Sherub Sherub, Henrik Skov, Tuvshintugs Sukhbaatar, Ramunas Zydelis, Martin Wikelski, Walter Jetz, Ivan Pokrovsky

**Affiliations:** ^a^Center for Biodiversity and Global Change, Yale University, New Haven, CT 06511; ^b^Department of Ecology and Evolutionary Biology, Yale University, New Haven, CT 06511; ^c^Bren School of Environmental Science and Management, University of California Santa Barbara, Santa Barbara, CA 93117; ^d^Department of Migration, Max Planck Institute of Animal Behavior, Radolfzell 78315, Germany; ^e^Schmalhausen Institute of Zoology of the National Academy of Sciences of Ukraine, Laboratory of Ornithology of the South of Ukraine, Kyiv 01054, Ukraine; ^f^Wildlife Science and Conservation Center of Mongolia, Ulaanbaatar 14210, Mongolia; ^g^Nature Research Centre, Vilnius 08412, Lithuania; ^h^BirdLife Denmark, Copenhagen 1620, Denmark; ^i^Institute of Zoology, Ministry of Science and Higher Education of the Republic of Kazakhstan, Almaty 050060, Kazakhstan; ^j^Daurskii State Nature Biosphere Reserve, Nizhny Tsasuchei, Transbaikalia 674495, Russia; ^k^Institute of Natural Resources, Ecology, and Cryology, Siberian Branch, Russian Academy of Sciences, Chita, Transbaikalia 672014, Russia; ^l^Severtsov Institute of Ecology and Evolution, Russian Academy of Sciences, Moscow 119071, Russia; ^m^Grimsö Wildlife Research Station, Department of Ecology, Swedish University of Agricultural Sciences, Riddarhyttan, Riddarhyttan S-730 91, Sweden; ^n^Vavilov Institute of General Genetics, Russian Academy of Sciences, Moscow 117971, Russia; ^o^Ugyen Wangchuck Institute for Forestry Research and Training, Bumthang 32001, Bhutan; ^p^Ecology and Environment Department, DHI, Hørsholm 2970, Denmark; ^q^Ornitela, Vilnius 03228, Lithuania; ^r^Department of Biology, University of Konstanz, Konstanz 78315, Germany

**Keywords:** migration, ecological niche, animal movement, life history

## Abstract

Movement is a key means by which animals cope with variable environments. As they move, animals construct individual niches composed of the environmental conditions they experience. Niche axes may vary over time and covary with one another as animals make tradeoffs between competing needs. Seasonal migration is expected to produce substantial niche variation as animals move to keep pace with major life history phases and fluctuations in environmental conditions. Here, we apply a time-ordered principal component analysis to examine dynamic niche variance and covariance across the annual cycle for four species of migratory crane: common crane (*Grus grus*, n = 20), demoiselle crane (*Anthropoides virgo*, n = 66), black-necked crane (*Grus nigricollis*, n = 9), and white-naped crane (*Grus vipio*, n = 9). We consider four key niche components known to be important to aspects of crane natural history: enhanced vegetation index (resources availability), temperature (thermoregulation), crop proportion (preferred foraging habitat), and proximity to water (predator avoidance). All species showed a primary seasonal niche “rhythm” that dominated variance in niche components across the annual cycle. Secondary rhythms were linked to major species-specific life history phases (migration, breeding, and nonbreeding) as well as seasonal environmental patterns. Furthermore, we found that cranes’ experiences of the environment emerge from time-dynamic tradeoffs among niche components. We suggest that our approach to estimating the environmental niche as a multidimensional and time-dynamical system of tradeoffs improves mechanistic understanding of organism–environment interactions.

## Significance

Animals must contend with spatially heterogeneous and temporally varying environmental conditions. Animals move to selectively modify the set of environmental conditions to which they are exposed. These movements produce multidimensional and time-varying “individual niches” which are expected to covary with animals’ schedule of life history events such as breeding or migration. Here, we characterize individual niche variation for four species of migrant crane and find that temporal variation in environmental niche is linked to species-specific life history events and migratory movements. Our approach offers a tractable, data-driven framework for identifying dynamic and transitory niche associations which are important for understanding how animals survive in complex environments.

Movement is a primary means by which animals cope with heterogeneous and temporally variable (i.e., dynamic) environments (1, 2). By moving, individual animals dynamically modify the set of environmental conditions to which they are exposed—their “individual niche” (3–5). An animal’s individual niche represents the subset of the species’ realized niche (6, 7) corresponding to the conditions experienced by the individual and can be estimated from the (conceptually similar) environmental utilization distribution (8, 9). Frequently, these movements arise from preference for favorable conditions (10, 11) or avoidance of unfavorable conditions (12), thus dampening the amplitude of resource variation over time (13). However, because the individual environmental variables comprising the niche (hereafter “niche components”) exhibit covariance relationships across space and time, individual niche dynamics may be more complex than simply seeking favorable conditions or avoiding unfavorable ones.

**Fig. 1.**
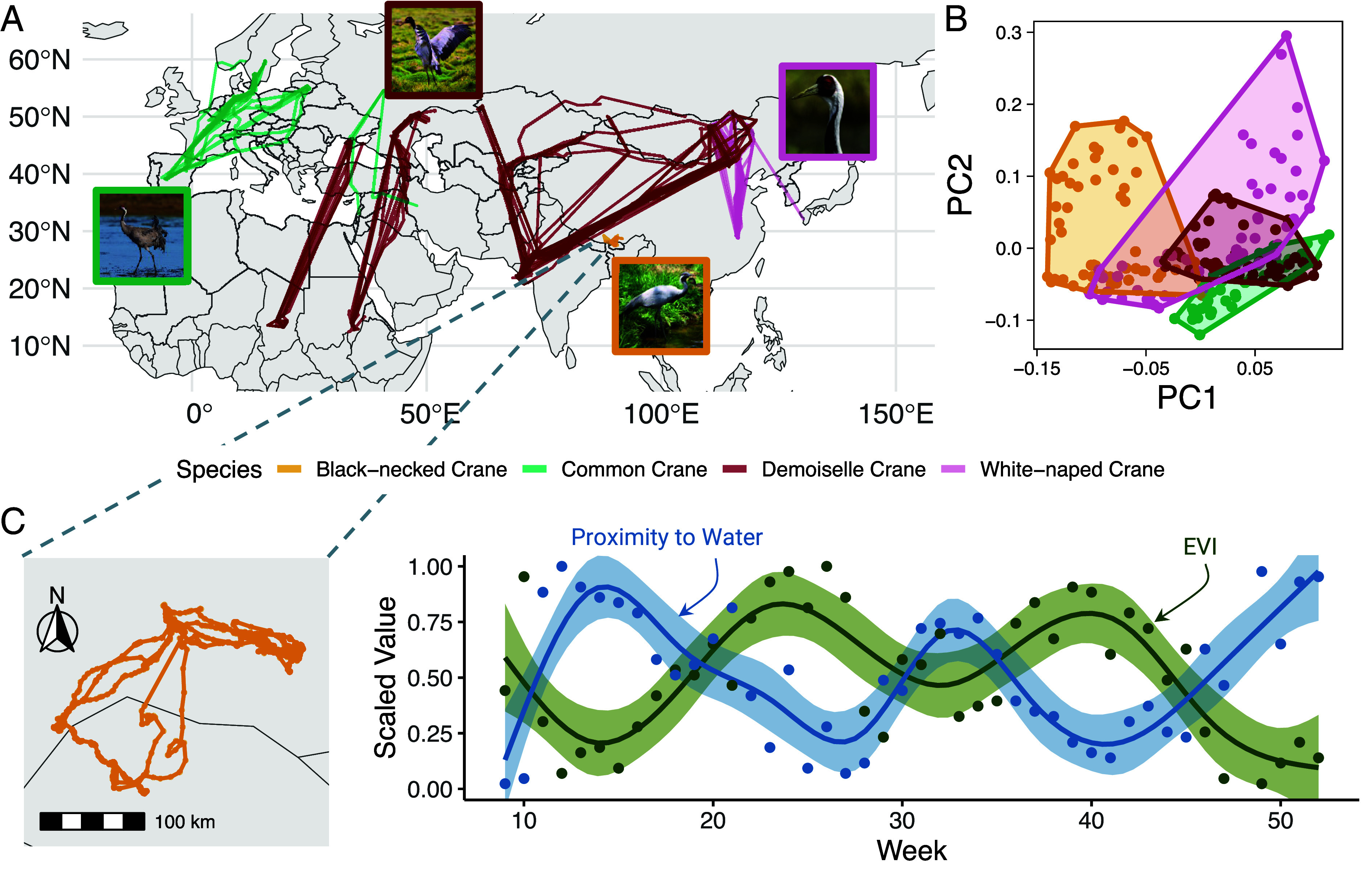
(*A*) GPS tracks from 104 individuals of 4 species of crane spanning Africa, Asia, and Europe: common crane (*G. grus*; green, n = 20), demoiselle crane (*A. virgo*; red; n = 66), black-necked crane (*G. nigricollis*; orange, n = 9), and white-naped crane (*G. vipio*; pink, n = 9). (*B*) Traditional PCA among species reduces dimensionality of environmental variables (here water proximity, proportion crops within 300 m, EVI, and land surface temperature; see *Materials and Methods*) but ignores within-species tradeoffs among variables (niche components) and temporal dynamics. Points represent weeks. (*C*) GPS tracks and time series of two niche components (water proximity and EVI) for a single individual black-necked crane suggest tradeoffs between proximity to risk (low values of water proximity suggest high predation exposure) and reward (high EVI indicates relatively high primary productivity). Values are scaled to individual-specific empirical quantiles.

Interactions between particular niche components can force animals to engage in compromises. Components of an organism’s niche may conflict with one another (Fig. 1*C*), forcing tradeoffs between competing niche components (14, 15). In such cases, organismal responses should reflect an optimized balance between the two environmental pressures (16, 17). For example, female elk (*Cervus elaphus*) with calves in Yellowstone National Park increased the frequency of predator vigilance behaviors at the expense of foraging efficiency in the proximity of gray wolves (*Canis lupus*; 15). Animals may also resolve conflicts among niche components by dynamically switching environmental exposure over space and time as biological or ecological relevance fluctuates (18). After fledging, piping plover (*Charadrius melodus*) broods move toward more suitable foraging habitat and away from safer, but resource-poor, nest sites (19). In other words, animals might avoid direct conflicts between niche components altogether and instead mediate them dynamically over time in association with specific life history events.

Studies that consider niches as both multidimensional and dynamic (e.g., ref. 20) remain challenging to perform. At the species level, multidimensional niche analyses are common (Fig. 1*B*; 21–23) but often do not consider temporal dynamics in niche geometry (but see e.g., refs. 24–26). Conversely, animal movement studies commonly treat environmental associations of individuals or groups as dynamic, especially for migratory animals (5, 10, 27–30). However, incorporating dynamic *covariance* relationships among multiple variables remains analytically challenging. Including covariance relationships among components when describing an individual niche has the potential to elucidate tradeoffs among competing interests and reveal how animal behavior links to environmental conditions.

Highly vagile species may be particularly useful to study how animals dynamically mediate variance within and covariance among niche components because they are able to access diverse environments which do not co-occur in space and/or time. Seasonal migration, in particular, represents a movement in response to resource dynamism, frequently described as an adaptation to enable resource tracking (5, 10, 27, 28, 31). However, migration might not always lead to niche tracking. Indeed, within migratory birds, it has long been known that environmental associations can vary between winter and summer stationary periods (30, 32–34) along with, for example, behavior (35, 36), physiology (4), and morphology (36, 37). It has also been shown that some migrations modulate tradeoffs among niche components. For example, Humpback Whales (*Megaptera novaeangliae*) switch emphasis from tracking abundant food resources as they migrate from cold, high-latitude sites to emphasis on juvenile development and survival at low-latitude areas with warm waters (38, 39). Despite their long-distance movements, migrants may also experience high niche component variance during certain periods of the year, such as migratory journeys when individuals emphasize movement speed or efficiency (5). Thus, the breadth of the niche, not just its position, may also vary.

**Fig. 2.**
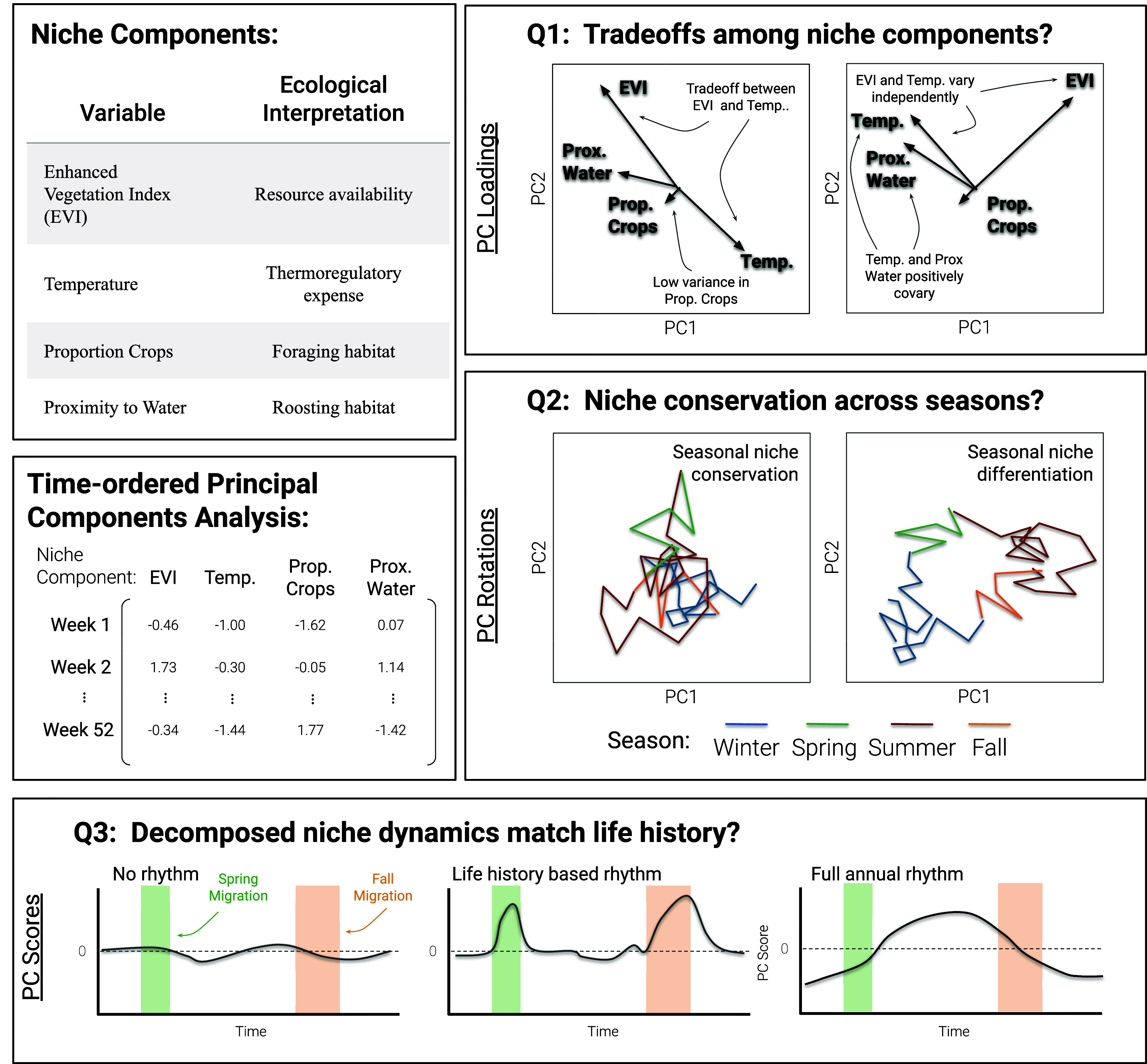
We considered four “niche components” (*Top* *Left*) with a priori ecological interpretations relevant to crane natural history. By organizing data as a weekly time series (*Middle* *Left*) before dimension reduction via PCA we can ask three questions about time-dynamic relationships among variables. (Q1, *Top* *Right*) Are there tradeoffs among niche components over the course of a full annual cycle? Opposing PC loadings indicate negative covariance (a tradeoff) among niche components, whereas concurring and orthogonal vectors imply positive covariance and independent covariance, respectively. Vector length indicates relative variance compared to other niche components; thus, very short vectors suggest niche components that do not vary strongly. (Q2, *Middle* *Right*) Are environmental niches conserved across seasons? By rotating the original data points back into environmental space (i.e., PC space) we can view the degree of overlap in niche axes among seasons. Seasons with little or no overlap imply use of different niches between seasons. (Q3, *Bottom*) How do particular axes of variation (PCs) contribute to variance in niche components over time? By plotting PC scores over time, we observe the relative degree to which each week of data contributes to overall variation in that axis. Scores close to zero imply very little variance in that week, whereas scores deviating from zero suggest large (positive or negative) variance contributed by that particular week and reveal how particular axes of niche variation associate with major phases of life history (e.g., migrations).

Here, we develop a data-driven framework for simultaneously treating individual niches as multidimensional and dynamic, while also explicitly estimating dynamic covariance among niche components (Fig. 2). We characterize the dynamics of niche components for four species of an iconic migratory clade: cranes (Gruidae), two of which are of conservation concern (40). The four species in this study exhibit different migratory strategies and species-level realized niches (Fig. 1*A*; 41–44). Additionally, individual tracking data suggest the potential for tradeoffs among key niche components (Fig. 1*C* and *SI Appendix*, Fig. S1). We consider four primary “Grinellian” niche components (45) for all species, each with well-documented relevance to crane natural history (Fig. 2). Two reflect important habitats: proportion of crops (“crop proportion,” preferred foraging habitat) and proximity to water (“water proximity,” preferred roosting habitat). We also consider two niche components which are broadly associated with migratory dynamics across taxa: temperature and a proxy for environmental gross primary productivity. Using a time-ordered principal component analysis (PCA) applied to movement data (Fig. 2), we are able to describe multidimensional dynamics in these four environmental variables, including dynamic patterns of covariance among niche components, as experienced by cranes. Specifically, we address 3 basic questions (Fig. 2):

1. Do cranes trade off among niche components across the annual cycle?
2. Are crane’s individual seasonal niches conserved (tracked) across seasons?
3. What are the temporal “rhythms” of niche dynamism and how are they linked to biologically relevant periods of the annual cycle?

The individual niche dynamics we uncover highlight the role of spatial movements in modulating covariance among environmental conditions. Our approach offers a simple and computationally tractable data-driven framework for addressing organism–environment interactions in heterogeneous landscapes.

## Results

We tracked 104 individuals of four species: common crane (*Grus grus*, n = 20), demoiselle crane (*Anthropoides virgo*, n = 66), black-necked crane (*Grus nigricollis*, n = 9), and white-naped crane (**Grus* vipio*, n = 9; Fig. 1). In our analysis, the first two PCs explained at least 89% of the variance in annual niche position (*SI Appendix*, Table S1) and breadth (*SI Appendix*, Table S2) for all species. Therefore, we only considered the first two PCs in subsequent analyses.

### Niche Component Tradeoffs.

**Fig. 3.**
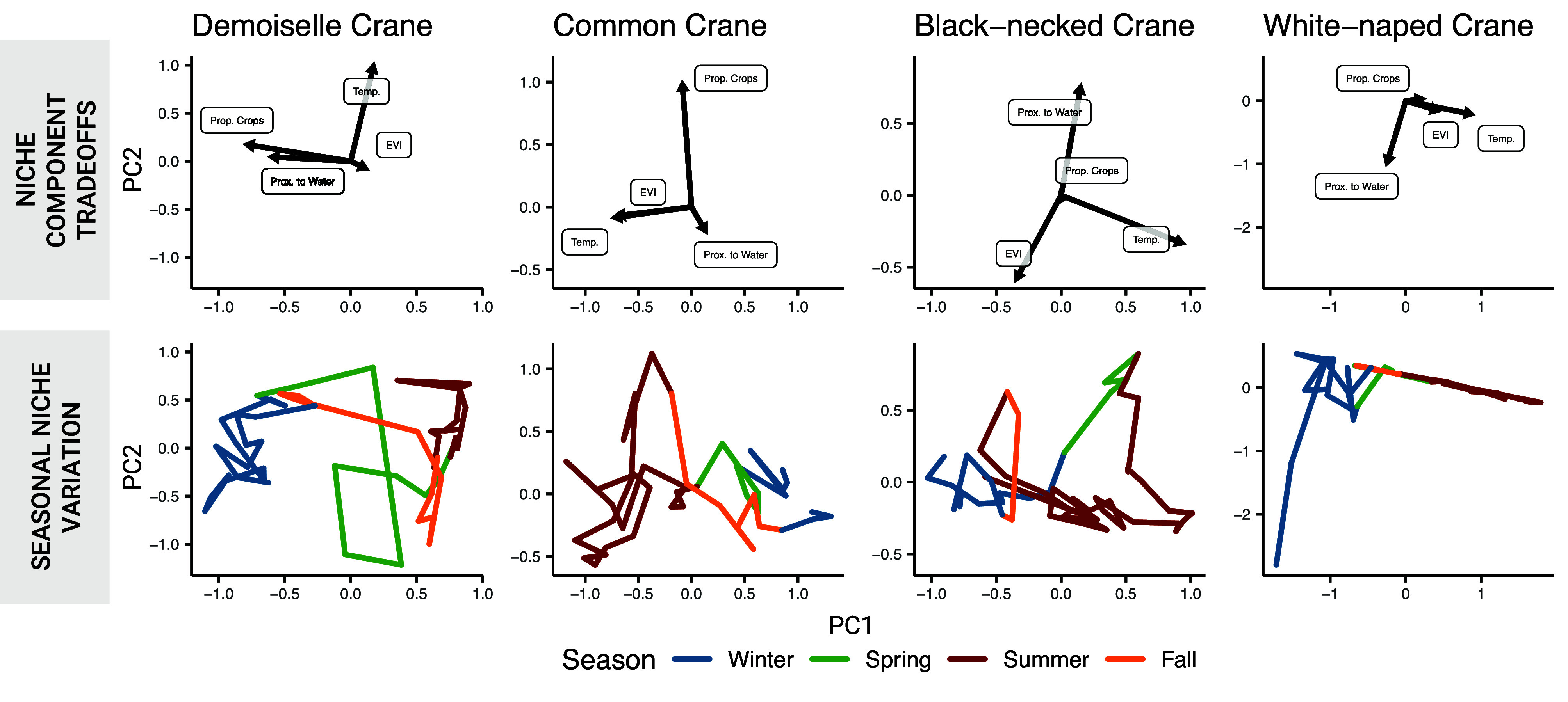
(*Top* row) PC loadings for each species reveal patterns of covariance among niche components over the course of an annual cycle. Orthogonal loadings imply uncorrelated variance, suggesting modulation of niche components across the annual cycle rather than direct tradeoffs. Opposing loadings suggest a tradeoff among niche components (as in black-necked crane between water proximity and EVI). Shorter vectors suggest low variance in a niche component over the course of a year. (*Bottom* row) Species’ seasonal niche variation plotted using the first 2 PCs. Niches are differentiated when there is little overlap in environmental space across seasons (e.g., summer and winter in demoiselle crane). Niches are consistent between seasons (tracked) when overlapping in PC space (e.g., spring and fall in white-naped crane).

We assessed evidence of tradeoffs among niche components (indicated by opposing PC loadings; Fig. 2, Q1) and found little evidence of niche component tradeoffs over the annual cycle in either niche position or breadth (Fig. 3 and *SI Appendix*, Fig. S2). Only black-necked crane showed a tradeoff between water proximity and enhanced vegetation index (EVI) as evidenced by opposing loadings (Fig. 3); no other opposing loadings were observed. Instead, all other loadings among niche components suggested positive covariance, independent variance, or little to no contribution to variance. For example, for demoiselle cranes, crop proportion and water proximity positively covaried, whereas temperature was independent of those niche components (Fig. 3). Similarly, in common crane and white-naped crane, temperature and EVI positively covaried, whereas crop proportion and water proximity (respectively) varied independently (Fig. 3).

In each species, at least one niche component was relatively stable across the annual cycle (as indicated by a short loading vector), specifically, crop proportion in black-necked crane and white-naped crane, and water proximity in demoiselle crane and common crane (Fig. 3). This was also true with respect to niche breadth: EVI in demoiselle crane and white-naped crane, crop proportion in black-necked and common crane, and temperature for all four species.

### Seasonal Niche Tracking.

We determined whether cranes tracked their niches by assessing overlap in seasonal niches in PC space (Fig. 2, Q2). Three out of the four species (demoiselle crane, common crane, and white-naped crane) showed complete niche position differentiation between winter and summer (no overlap between seasons in environmental space; Fig. 3), and the black-necked crane only had minor overlap. For the same three species, migratory niche positions were intermediate between the two seasons (Fig. 3). Migratory periods in black-necked crane overlapped summer, indicating conservation of a single niche from spring to fall in the species. Only two of the four species showed seasonal differences in niche breadth: demoiselle crane and white-naped crane (*SI Appendix*, Fig. S2).

Spring and fall niches were less clearly differentiated from one another than were the two stationary periods. Only black-necked crane showed complete separation in niche position between spring and fall migration, whereas the migratory periods were nearly completely overlapping in the other three species (Fig. 3). Similarly, only demoiselle crane showed differences in niche breadth between spring and fall migration while the other three species exhibited substantial overlap among seasons (*SI Appendix*, Fig. S2).

### Niche Dynamics.

**Fig. 4.**
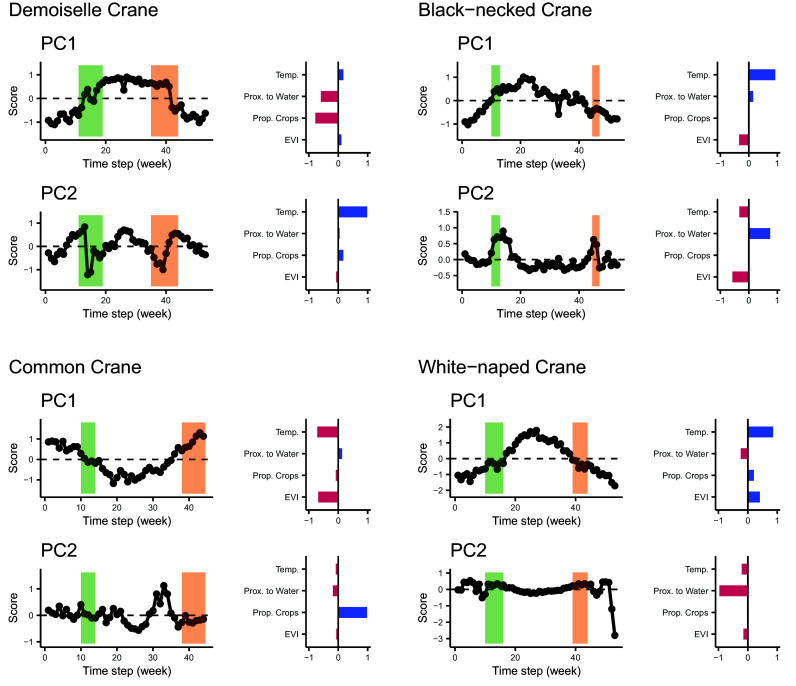
Annual niche dynamics and their components. Line plots show PC scores per week (i.e., per sample) for the first two PCs in relation to spring and fall migration (green and orange shaded areas, respectively). Values that deviate from zero indicate weeks that contributed relatively more to that PC. Bar plots show niche component loadings on that PC. Similar to the scores, niche component loadings that deviate from zero indicate greater relative contribution of a certain covariate to that PC. Temporal trends in PC scores reveal seasonal patterns of variation in the niche components indicated by the bar plots. For example, for demoiselle crane, PC1 is composed almost entirely of water proximity and crop proportion and exhibits a temporal trend broadly matching patterns of seasonality. On the other hand, PC2 for that species shows strong variation during spring and fall migration but less so during winter or summer stationary periods. This migration-associated PC is driven primarily by variance in temperature.

We characterized the major rhythms of niche variation using the time series of PC scores along with axis-specific loadings (Fig. 2, Q3). For niche position, in all species, winter and summer PC1 scores were the inverse of one another and gradually transitioned between those states (Fig. 4). However, the different loadings of PC1 mean that the niche components underpinning this rhythm were species specific. Seasonal changes in individual niche position were primarily associated with water proximity and crop proportion in demoiselle crane, temperature and EVI in common crane and black-necked crane (but positively and negatively covarying with each other, respectively), and temperature, water proximity, and EVI in white-naped crane (Fig. 4).

The secondary niche position rhythm (PC2) in demoiselle crane, black-necked crane, and white-naped crane appeared to be associated with both spring and fall migratory periods (Fig. 4). In demoiselle crane, this rhythm was almost exclusively associated with variance in temperature, whereas in black-necked crane and white-naped crane, migration niche variance involved all niche components except crop proportion. The secondary niche position rhythm for common crane appeared to be related to niche dynamics within the breeding season. PC2 scores delineated an early and late breeding season associated almost exclusively with variance in the proximity to crops.

We also found evidence of niche breadth variation associated with life-history events (*SI Appendix*, Fig. S3). Demoiselle and white-naped cranes exhibited inverted PC1 scores (linked to water proximity and proportion crops) between winter and summer but, unlike niche position, showed a rapid (rather than gradual) transition between those states. The primary individual niche breadth rhythm for common and black-necked cranes showed brief spikes during fall (common crane) or both (black-necked crane) migration periods. The secondary rhythm was almost entirely associated with differences in EVI variance between the late winter and early breeding periods for common cranes, whereas this rhythm was far noisier in white-naped crane (though may show migration-associated changes in niche breadth). PC2 explained a negligible proportion of the variance for demoiselle crane and black-necked crane (*SI Appendix*, Table S2).

## Discussion

We found that cranes’ migratory movements largely avoided direct tradeoffs among niche components (both position and breadth) over the course of the annual cycle. Instead, niche associations varied seasonally and were synchronized with key phases of life history. Thus, by considering multiple niche components simultaneously, we show how animal movement within temporally varying and heterogeneous environments produces complex patterns of individual niche covariance that are specifically linked to key phases in species’ life histories. Specifically, our results suggest that temporal dynamics may differ among niche components resulting from idiosyncratic relationships between particular environmental conditions and specific aspects of organismal biology. In this way, animal movements may result in tracking constant conditions with respect to some niche components but variable conditions with respect to others (Fig. 4), including differences among niche components in breadth (*SI Appendix*, Fig. S3). Niche component dynamism could plausibly arise in several ways: 1) It may be that species are simply agnostic to or tolerant of certain niche components, especially when non-movement adaptations facilitate persistence in variable environments such as those that experience strong seasonality (46, 47). 2) Niche tracking may be imperfect such that the magnitude of variance is reduced as compared to nontracking, but still results in variance over time. This dampening, but not elimination, of seasonal environmental variation has been recorded in multiple migrant species (48, 49). 3) Preferred conditions may only be periodically relevant and/or niche component preferences may not be stationary over time (50, 51). For example, in our study, common cranes showed subseasonal emphasis on the crop proportion during only the second half of the summer breeding season (a season wherein many migrant birds emphasize brood rearing and/or preparation for migration). It is worth noting that there may also be ontogenic shifts in niche preferences over time (52) which could be the target of future research.

Migrant cranes in our study appeared to largely avoid tradeoffs among niche components over the annual cycle with respect to both niche position and breadth. Only black-necked crane exhibited a tradeoff between resource availability (EVI) and predator avoidance (water proximity), primarily associated with spring and fall migratory periods (Fig. 3). Interestingly, and in contrast to the other three species in our study, black-necked crane is the only short-distance elevational migrant, whereas the other three species undertake longer-distance migrations. Thus, it may be that the species’ shorter migrations cannot resolve tradeoffs to the degree that the other long-distance migrants can. Future comparative work could consider the role of migration distance or elevation in mediating potential resource conflicts.

We found little evidence of seasonal individual niche tracking in the four species of crane examined. Seasonal migrations have been shown to produce both niche tracking (5, 27, 53) and niche switching dynamics (48, 49, 54). In this study, season-specific environmental niche positions were predominantly nonoverlapping (Fig. 3). Moreover, the dominant rhythm of niche variation over time recapitulated the full annual seasonal cycle (including inverted variable relationships between summer and winter; Fig. 4). That being said, some niche components (position and breadth) were relatively invariant over the course of a year suggesting possible niche tracking with respect to particular niche components but not others (Fig. 3 and *SI Appendix*, Fig. S2).

We also observed niche dynamism that was specifically associated with life history events (e.g., migratory periods or intraseasonal dynamics, depending on the species). For example, 29.5% of niche position variation (PC2, *SI Appendix*, Table S1) in demoiselle cranes was almost solely associated with dramatic reductions in temperature during migratory periods (Fig. 4), likely resulting incidentally from individuals crossing the Tibetan Plateau rather than specifically tracking low temperatures (55). In fact, crossing geographic barriers during migration may be a significant cause of niche variance in cranes more generally (52, 56, 57). We include these brief periods of niche configuration in our discussion of the individual niche because surviving those transits is necessary for individuals to access the geographic locations which provide more hospitable conditions. Interestingly, niche breadth also appeared to vary in this species between the two stationary periods (with the migrations intermediate between the two) but did not show a fall migration-specific change.

The PCA-based framework we used here has potential for future extensions. It has previously been used to uncover biogeographic patterns in functional trait diversity (58) but has otherwise been little used in ecology, despite potential applications across several subdisciplines. For example, there is growing appreciation that aspects of the human environment influence animal movements (59–62). Because time-ordered PCA is well suited to capture dynamic covariance among variables, future work might aim at understanding how aspects of the human environment relate to other components of species’ niches to disentangle how anthropogenic effects modify species–environment interactions. Similarly, this framework could be extended to behavioral ecology to explore the dynamic structure of covariance among environmental and behavioral variables. This method also provides a data-driven method for identifying transient dynamics in organism–environment interactions which could support targeted conservation or management actions by revealing important, but short-lived, resource associations that are currently difficult to characterize with other methods. It is important to note that individual PC axes can be translated back into resource values using axis-specific scores and loadings. Doing so could isolate niche dynamics associated with particular life history phases of conservation or management interest. In this study, we used simple seasonal phases to capture major life-history events, but future work could consider individual niche dynamics across alternative characterizations of life history events (e.g., molting, courtship). Our method could also be paired with canonical resource selection analyses (63) to first reveal important variable combinations associated with periods of interest using time-ordered PCA followed by a comparison with local availability to characterize preference for and against resources in a spatially explicit framework. Finally, this approach could be extended to include among-individual heterogeneity in, for example, niche dynamics (20, 64) and/or behavior (65, 66).

Treating species’ niches as dynamical systems with complex patterns of variance and covariance may prove useful for understanding and predicting the impacts of global anthropogenic environmental change. Previous work, mostly in single-species systems, has highlighted complicated and dynamic relationships between certain environmental factors and specific organismal outcomes (67). More specifically, population biology has long recognized that environmental factors can interact uniquely with particular fitness components or stages of the reproductive cycle (68). Including the potential for idiosyncratic, dynamic, and occasionally transient relationships between organisms and particular niche components may improve predictions about species’ responses to changing environments (26, 69–71). Specifically, the PCA method presented here could be used as an initial hypothesis-generating step to reveal environmental drivers potentially associated with population processes. For example, the late breeding season emphasis on crop proportion we observed in common cranes may suggest a link between resource availability and juvenile survival which could be the target of future demographic analyses.

Our work highlights the complicated and behaviorally mediated environmental dynamics which organisms experience. Future extensions focused on linking these dynamics to demographically relevant outcomes would provide critically important insights into the mechanistic basis for population persistence and conservation-relevant characterizations of organism–environment relationships. Given dramatic declines in abundance across taxa (72–75), and among migrants in particular (74, 76), developing approaches that mechanistically link environmental conditions to population outcomes remains a pressing need.

## Materials and Methods

### Data Collection.

We developed a system to track white-naped, black-necked, and demoiselle cranes with solar-powered leg bands that collect GPS information. Tags were manufactured at the Max Planck Institute of Animal Behavior, Department of Migration, and sent to collaborators in the field. Detailed methodologies are described in refs. 52 and 55–57. Common Crane data were obtained from existing studies stored on Movebank; detailed methodology can be found in ref. 77. The original tracking data used in this study are publicly available (see *Data, Materials, and Software Availability*). Between 2011 and 2021, we tracked individuals of four different species: common crane (*G. grus*, n = 20), demoiselle crane (*A. virgo*, n = 66), black-necked crane (*G. nigricollis*, n = 9), and white-naped crane (*G. vipio*, n = 9; Fig. 1*A*).

### Data Preparation and Annotation.

To remove outliers and potentially erroneous positions, we excluded observations exhibiting extreme turn angles or step lengths. Any observations where the inbound step or turn angle exceeded the individual-specific 95% quantile were removed. Because datasets were gathered with heterogeneous technologies, on-board positional accuracy measurements differed across individuals. When applicable, we excluded observations with a recorded dilution of precision (DOP) or horizontal DOP ≥5 or horizontal accuracy ≥25 m. We also excluded any individuals whose total track duration was <50 d. This resulted in a final dataset of 104 individuals: 20 common crane (*G. grus*), 66 demoiselle crane (*A. virgo*), 9 black-necked crane (*G. nigricollis*), and 9 white-naped crane (*G. vipio*).

To facilitate analyses across a large dataset, we organized data in an SQLite relational database using the open source *mosey_db* tool (https://benscarlson.github.io/mosey). We annotated each observation from the cleaned dataset with four environmental variables: proportion of crops, water proximity, land surface temperature, and EVI. Crop proportion in the vicinity of each observation represents the availability of high-quality foraging habitat. Proportion of crops was calculated within a 300 m buffer surrounding each observation using the European Space Agency Climate Change Initiative Land Cover time-series [300 m spatial resolution, annual temporal resolution; (78)]. Cranes typically roost in open, shallow water to reduce the risk of predation. Therefore, we annotated each observation with the distance to the nearest water body which we calculated using a Landsat-based dynamic surface water dataset [30 m spatial resolution, monthly temporal resolution; (79)]. We also annotated observations with MODIS Daily Land Surface Temperature (MODIS/006/MOD09GA; 500 m spatial resolution, daily temporal resolution) and MODIS EVI (MODIS/MOD09GA_006_EVI; 1 km spatial resolution, daily temporal resolution). Temperature is associated with thermoregulatory expense, and both temperature and EVI are associated with primary productivity and, ultimately, prey availability. Environmental annotations were completed using the open-source *mosey_env* tool (https://github.com/benscarlson/mosey_env), which is a companion to *mosey_db* that leverages Google Earth Engine via the *rgee* package (80) in R (81), except for the landcover annotations which used the Map of Life STOAT annotation tool (82).

### Time-Dynamic PCA.

We used a time-dynamic approach to PCA to recover major tradeoffs among niche components, assess niche conservatism across seasons, and characterize the rhythms of covariance among niche components (Fig. 2). Time-dynamic PCA builds upon the canonical PCA approach while preserving the time-ordered structure of the data. Thus, the scores and rotations of the original data retain information about the temporally explicit structure of variance/covariance within the dataset.

To prepare data for species-level PCA, we summarized individual observations to a weekly resolution for each species. To do so, we first calculated individual-specific weekly means for each of the four niche components. Thus, the data for each individual crane are summarized to one value per week for each of the four niche components. Then, for each week, we calculated the among-individual median for each of the four niche components. This process resulted in one value per niche component summarizing niche position in each week of the year, stored as a 53 × 4 matrix for each species (44 rows in the case of common crane), where rows correspond to the weeks of the year, and columns correspond to the 4 niche components. These values can be interpreted as estimates of species-level median individual niche position. We then performed canonical PCA using the *prcomp* function in R. We also considered dynamic covariance in weekly individual niche breadth. To do so we repeated the process above but used individual-specific weekly variance (rather than mean) for each of the four niche components such that the final data supplied to the PCA analysis represented species’ weekly median individual niche breadth.

We interpret the overall PC loadings to assess evidence of tradeoffs among niche components over the annual cycle (Fig. 2, Q1). Orthogonal loadings suggest independent variation among niche components, whereas opposing loadings imply negative covariance and, thus, a tradeoff. Vector length represents a relative contribution to overall variance; short vectors suggest constancy (tracking) with respect to that niche component. Rotating the original environmental data according to the PCA can be interpreted as the species’ average “track” in environmental space (rather than geographic space; Fig. 2, Q2). Using the rotated data, we assessed whether cranes tracked environmental niches across seasons by evaluating interseasonal overlap in PC space. Finally, the sample-wise PC scores for each axis reveal the temporal “rhythms” of variation for that specific PC (Fig. 2, Q3). Scores near zero imply very little contribution of that sample (in this case week) to the covariance explained by that particular PC, whereas scores deviating from zero imply the converse. We visualized PC scores as a time series in concert with major life history phases (winter residency, migratory periods, and summer breeding) to determine how particular PCs related to species’ life histories.

### Migration Phenology.

We identified transitions between the four major phases of the annual cycle (winter residency, summer breeding, spring migration, and fall migration) manually for each individual in our dataset. We inspected a combination of maps of observations in geographic space, time series plots of latitude and longitude (separately), and a time series plot of net squared displacement. We manually identified transitions between winter or summer stationary periods and the adjacent migratory periods. In cases where transitions were uncertain, we added a quality control flag to denote this uncertainty and excluded those individuals from summary analyses. We calculated species-level transition dates as the median transition date among individuals.

## Supplementary Material

Appendix 01 (PDF)

## Data Availability

The GPS track data used in this study have been deposited in the Movebank Data Repository (83–92). All code used to perform analyses and produce visualizations associated with this manuscript are publicly available at Zenodo (https://doi.org/10.5281/zenodo.13530118) (93) or Github (https://github.com/syanco/cranes_niches) (94).
