# Supplementary material for: Migratory birds modulate niche tradeoffs in rhythm with seasons and life history
Source: Proc Natl Acad Sci U S A. 2024 Sep 23;121(41):e2316827121. doi: 10.1073/pnas.2316827121 (PMC11474074; doi:10.1073/pnas.2316827121)
Supplement: Supplementary file 1 — Appendix 01 (PDF) [file pnas.2316827121.sapp.pdf]

Supplementary Material for *Migratory birds modulate niche tradeoffs in rhythm with seasons and life history*

Table S1. Percent of variance explained by principal component analysis of niche position for the first two components for four species of crane.

Species	Percent Variance Explained		
	PC1	PC2	*Cumulative: PC1 & PC2*
Anthropoides virgo	62.2%	29.5%	*91.7%*
Grus grus	71.4%	17.6%	*89.1%*
Grus nigricollis	72.8%	23.0%	*95.8%*
Grus vipio	72.1%	17.9%	*90.1%*

Table S2. Percent of variance explained by principal component analysis of niche breadth for the first two components for four species of crane.

**Species**	**Percent Variance Explained**		
	**PC1**	**PC2**	***Cumulative: PC1 & PC2***
Anthropoides virgo	89.2%	5.8%	*95.0%*
Grus grus	79.8%	15.1%	*94.9%*
Grus nigricollis	99.1%	0.5%	*99.6%*
Grus vipio	69.2%	20.0%	*89.2%*

Table S3. Sample size summary after data cleaning (see Methods) for four species of crane.

Species	No. Individuals	Mean Track Duration (weeks)	Mean Fixes per Week	Total Fixes
Anthropoides virgo	66	22.4	68.1	86,476
Grus grus	20	73.3	688	475,016
Grus nigricollis	9	100.8	382	156,224
Grus vipio	9	97.8	17.4	6,182

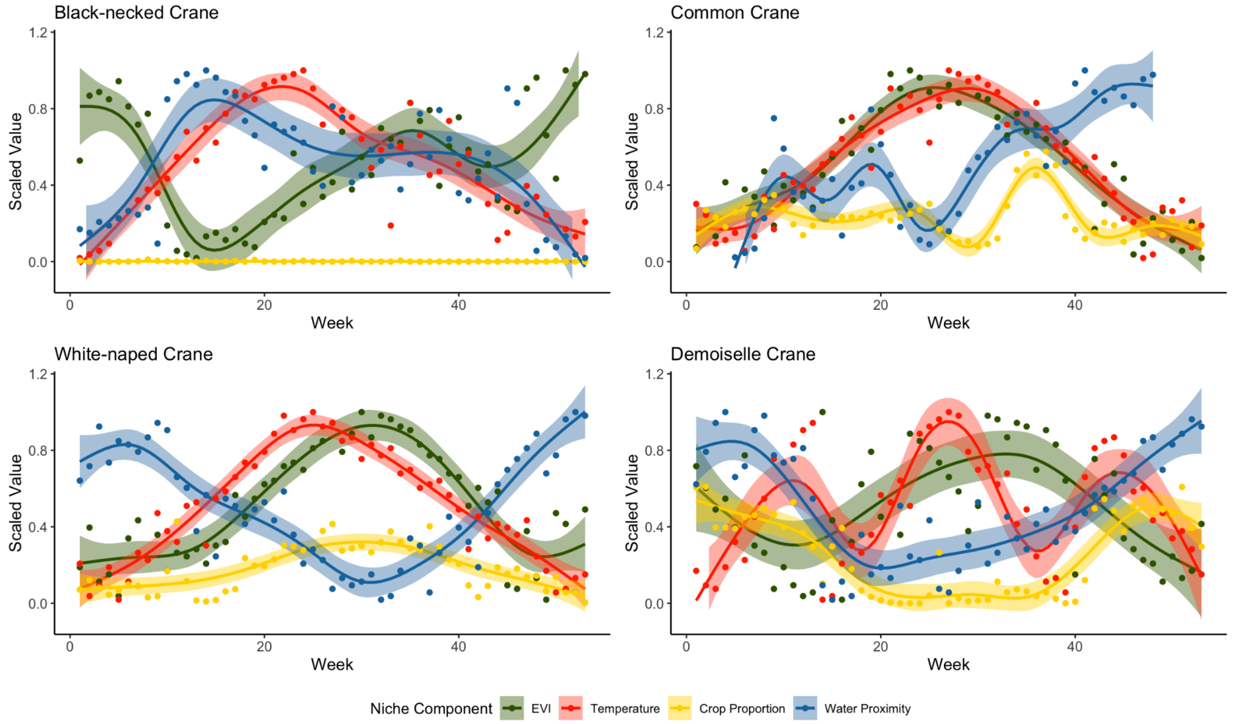

Figure S1. Time series of niche components for all species. Points represent among-individual mean of individual weekly means. Values are scaled to individual specific empirical quantiles.

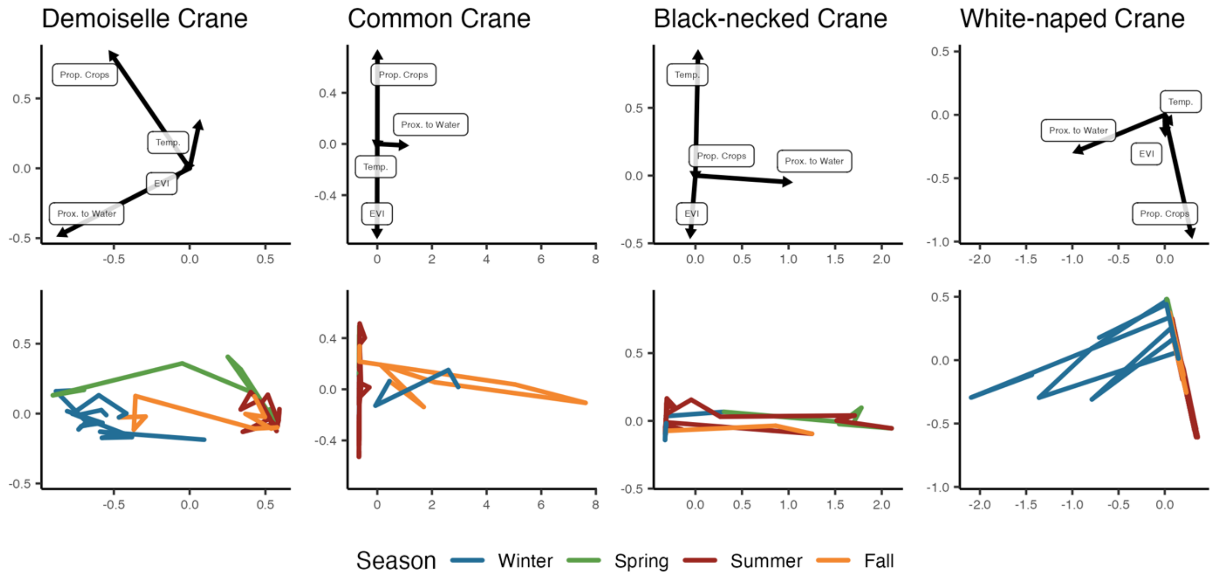

Figure S2. (Top row) Principal components loadings for each species reveal patterns of covariance among niche component breadths over the course of an annual cycle. Orthogonal loadings imply uncorrelated variance, suggesting modulation of niche component breadth across the annual cycle rather than direct tradeoffs. Opposing loadings suggest a tradeoff among niche component breadths (as in common crane between proportion crops and EVI). Shorter vectors suggest low variance in niche component breadth over the course of a year. (Bottom row) Species' seasonal niche breadth variation plotted using the first 2 principal components. Niche breadths are differentiated when there is little overlap in environmental space across seasons (e.g., summer and winter in demoiselle crane). Niches are consistent between seasons (tracked) when overlapping in PC-space (e.g., spring and fall in white-naped crane).

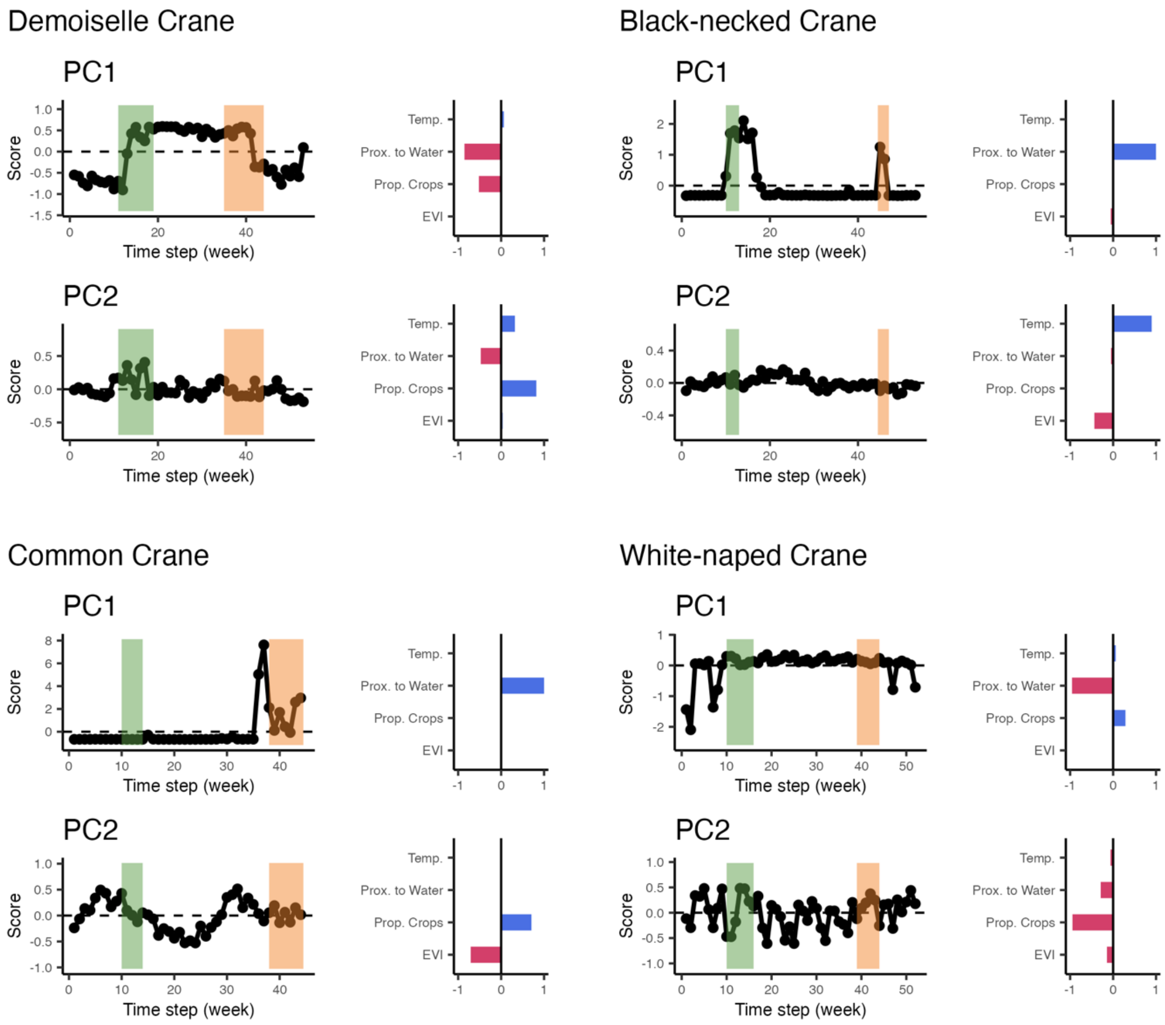

Figure S3. Annual niche breadth dynamics and their components. Line plots show principal component (PC) scores per week (i.e., per sample) for the first two PCs in relation to spring and fall migration (green and orange shaded areas, respectively). Values that deviate from zero indicate weeks that contributed relatively more to that principal component. Bar plots show niche component breadth loadings on that PC. Similar to the scores, niche component loadings that deviate from zero indicate greater relative contribution of a certain covariate to that PC. Temporal trends in PC scores reveal patterns of variation in the niche component breadths indicated by the bar plots. For example, for demoiselle crane PC1 is composed almost entirely of water proximity and crop proportion and appears to vary between breeding and wintering grounds (narrow on summer breeding grounds and wide during winter).
